# Advantage of laparoscopy surgery for elderly colorectal cancer patients without compromising oncologic outcome

**DOI:** 10.1186/s12893-020-00967-6

**Published:** 2020-11-23

**Authors:** Yih-Jong Chern, Hsin-Yuan Hung, Jeng-Fu You, Yu-Jen Hsu, Jy-Ming Chiang, Pao-Shiu Hsieh, Wen-Sy Tsai

**Affiliations:** 1 Division of Colon and Rectal Surgery, Department of Surgery, Chang Gung Memorial Hospital, Chang Gung University College of Medicine, No. 5, Fu-Hsing St, Kuei-Shan, Tao-Yuan, Taiwan; 2 School of Medicine, Chang Gung University, 259 Wen-Hwa 1st Road, Kuei-Shan, Tao-Yuan, Taiwan

**Keywords:** Colorectal cancer, Elderly, Laparoscopic surgery, Outcome

## Abstract

**Background:**

Laparoscopic surgery has achieved significant results in elderly patients with colorectal cancer (CRC). In this study, we compared the short-term and long-term outcomes of open surgery and laparoscopic surgery in patients with CRC aged above 75 years at a single tertiary medical center.

**Methods:**

We analyzed 967 patients who underwent curative resection for primary colorectal adenocarcinoma without distant metastasis between January 2009 and December 2015, in a single institution. Of the enrolled patients, 305 underwent laparoscopic surgery, and 662 received open laparotomy surgery.

**Results:**

Compared to the patients who underwent open surgery, those who received laparoscopic surgery had significantly shorter postoperative stay (10.3 vs. 13.5 days *p* < 0.001) and similar postoperative morbidity (*p* = 0.354) and mortality (*p* = 0.082). In the laparoscopy cohort, six of 305 patients were converted to open surgery and one died. The long-term overall survival, cancer-specific survival, and recurrence rate were similar between both cohorts in each stage.

**Conclusions:**

Laparoscopic surgery is suitable for elderly patients owing to shorter postoperative stay, similar long-term outcomes with open surgery, and acceptable low conversion rates. For long-term overall and oncological outcomes, the results of laparoscopic surgery were similar to that of open surgery in each TNM stage.

## Background

Colorectal cancer (CRC) is a common and life-threatening disease worldwide. Many population data presented that approximately 30–40% of the CRC cases occur in patients aged above 75 years [1–4]. In 2009, approximately 31.5% of the patients with CRC were aged above 75 years in Taiwan, with the number of patients increasing with the slowly aging population. However, some studies reported high morbidity and mortality in elderly patients undergoing open colorectal surgery [5, 6]. A systematic review of 28 studies also presented increase in postoperative morbidity and mortality with age [7].

It is well-established that increasing age is associated with increase in comorbidities, which further enhances surgical morbidity and mortality. Nevertheless, with a significant progress within the past two decades, laparoscopic surgery has emerged as an alternative for elderly patients. Randomized control trials like COST, CLASICC, COLOR and COREAN, and JCOG have demonstrated that although laparoscopic surgery has similar long-term outcomes and morbidity/mortality compared to open surgery, the short-term outcomes are better [8–12]. Laparoscopic surgery is beneficial as there is less operative pain, less blood loss, shorter hospital stay, and quicker recovery time [13]. However, it is limited by longer operation time and the potential cardiopulmonary change induced by pneumoperitoneum.

The aforementioned major randomized control trials compared laparoscopic surgery with open surgery; however, they did not focus on elderly patients. Many studies reported significant short-term outcomes and morbidity/mortality rate, enabling the elderly patients who received laparoscopic surgery to return to normal life within a short duration [13–15]. However, prospective studies comparing laparoscopic surgery with open surgery on long-term outcomes in elderly patients are rare. In this study, we aimed to identify whether these short-term outcomes could also improve the long-term oncologic benefits of elderly patients in the laparoscopic group. Our study retrospectively compared the short-term and long-term survival outcomes between the open surgery group and the laparoscopic surgery group in patients with CRC aged above or equal to 75 years at a single academic medical center.

## Methods

### Patients and variables

The patients aged ≥ 75 years who underwent curative radical resection for primary colorectal adenocarcinoma between January 2009 and December 2015, were included in this study. Patients who received local tumor excision, diagnosed with stage IV disease, or acquired emergency operation were excluded from this study. A total of 967 patients were enrolled for the analysis. Of the enrolled patients, 305 underwent laparoscopic surgery, and 662 received open laparotomy surgery. The decision of laparoscopic or open surgery was taken based on the physician and patient’s preferences. The penetration rate of laparoscopic surgery has gradually increased annually, owing to the improvement of laparoscopic facilities and equipment, and the increasing number of surgeons who select laparoscopic radical resection as the first choice of treatment for elderly CRC patients.

Detailed information regarding clinicopathological variables was retrieved from the Colorectal Section Tumor Registry of Chang Gung Memorial Hospital. This study was approved by the Institutional Review Board. This project was reviewed and approved by our institutional ethics committee (No. 201801090B0). The patient-related variables included age, sex, body weight, body height, body mass index (BMI), and the presence of illness were recorded. Patients’ health information such as incidence of hypertension, cardiac disease, cerebrovascular accident, asthma, diabetes mellitus, and liver cirrhosis were also collected. Blood analysis including carcinoembryonic antigen (CEA), hemoglobin (Hb), albumin, aspartate aminotransferase (AST), total bilirubin, and creatinine (Cr) was performed before operation. The tumor-related variables included tumor invasion depth (T stage), lymph node involvement (N stage), histologic subtype, histologic grade, tumor location, operation types, and the number of sampled lymph nodes.

### Follow-up and end points

Physicians in the same department of this institute adopted similar follow-up routines and adjuvant treatment protocols. All patients participated in a follow-up program that included outpatient visits every 3–6 months for physical examination and CEA tests, as well as chest X-ray, abdominal sonography or abdominal computed tomography, and colonoscopy every 1–3 years, postoperatively. The endpoint was death from any cause for overall survival (OS) and cancer-related death for cancer-specific survival (CSS). The recurrence of cancer was confirmed using histology of biopsy specimens, re-operation, or radiological studies. The time to recurrence was defined as the duration between the date of initial surgery and date of recurrence confirmation. Cumulative recurrence rate (CRR) was referred to as the cumulative probability of colorectal cancer recurrence occurring during follow-up. The prognosis was evaluated by OS, CSS, and CRR.

### Statistics

All analyses were performed using the Statistical Package for the Social Sciences, release 24.0 (IBM SPSS v.24). Clinicopathologic characteristics were compared using the chi-square test and Student’s t-test for continuous data. OS, CSS, and CRR were computed using the Kaplan–Meier method. Differences were estimated using the log-rank test. Statistical significance was set at P < 0.05.

## Results

A total of 967 elderly patients were included in this study, with a median follow-up duration of 42.1 months. Of the enrolled patients, 662 (68.5%) received open surgery and 305 (31.5%) underwent laparoscopic surgery.

The demographic data are presented in Table 1. There was no difference in age and sex ratio between the open surgery group and the laparoscopic group. The laparoscopic group showed a higher rate of being overweight or obese, and having comorbidities of hypertension. The open group showed significantly advanced Tumor-Node-Metastasis (TNM) stage, higher rate of abnormal preoperative serum CEA level, and hypoalbuminemia.

**Table 1:** Demographic data

Characteristic	Open (*n* = 662)	Laparoscopy (*n* = 305)	*P*
Age (y/o)	80.4 ± 4.2	80.4 ± 4.6	0.878
Sex (male)	369 (55.7)	166 (54.4)	0.703
Body mass index (kg/m^2^)			0.014
Underweight (< 18.5)	54 (8.3)	16 (5.2)	
Healthy (18.5–25)	406 (62.1)	170 (55.7)	
Overweight (25–30)	168 (25.7)	98 (32.1)	
Obese (> 30)	26 (4.0)	21 (6.9)	
Hypertension	384 (58.0)	205 (67.2)	0.006
Cardiac disease	113 (17.1)	60 (19.7)	0.326
Cerebral vascular disease	53 (8.0)	20 (6.6)	0.428
Asthma	34 (5.1)	14 (4.6)	0.717
Diabetes mellitus	171 (25.8)	74 (24.3)	0.602
Cirrhosis	11 (1.7)	6 (2.0)	0.737
TNM stage			0.003
0	8 (1.2)	8 (2.6)	
1	83 (12.5)	62 (20.3)	
2	294 (44.4)	129 (42.3)	
3	277 (41.8)	106 (34.8)	
CEA > 5 (ng/ml)	238 (36.0)	75 (24.6)	< 0.001
Hb < 10 (g/dL)	176 (26.6)	76 (24.9)	0.583
Albumin < 3.5 (g/dL)	155 (23.4)	45 (14.8)	0.002
AST > 34 (U/L)	50 (7.6)	26 (8.5)	0.602
Total bilirubin > 1.3 (mg/dL)	16 (2.4)	7 (2.3)	0.908
Cr > 1.27 (mg/dL)	141 (21.3)	79 (25.9)	0.113

Data are presented as n (%) unless otherwise indicated. *CEA* carcinoembryonic antigen, *Hb* hemoglobin, *AST* aspartate transaminase, *Cr* creatinine

The operative data is shown in Table 2. There was a significantly higher percentage of patients who underwent anterior resection in the laparoscopic surgery group, and segmental resection, subtotal colectomy, and Hartmann’s procedure in the open surgery group. The open group and the laparoscopic group had similar retrieved lymph nodes. Although the postoperative morbidity and mortality were similar between both cohorts, the postoperative hospital stay was 10.3 ± 8.5 days in the laparoscopic group, which was significantly shorter than that of 13.5 ± 9.4 days (*p* < 0.001) in the open group. There were six patients out of 305 patients (2.0%) who initially underwent laparoscopy, following which open surgery was conducted. As illustrated in Table 3, advanced pathology T stage was observed in the open cohort; however, the N stage, histology type, and histology grade between the two cohorts were similar.

**Table 2:** Operative data

Characteristic	Open (*n* = 662)	Laparoscopy (*n* = 305)	*P*
Tumor site			0.419
Right colon	196 (20.6)	84 (27.5)	
Left colon	264 (39.9)	115 (37.7)	
Rectum	202 (30.5)	106 (34.8)	
Operation types			0.048
Right hemicolectomy	174 (26.3)	80 (26.2)	
Left hemicolectomy	41 (6.2)	23 (7.5)	
Anterior resection	375 (56.6)	185 (60.7)	
Abdomino-peritoneal resection	16 (2.4)	8 (2.6)	
Segmental resection	13 (2.0)	0 (0)	
Subtotal colectomy	17 (2.6)	2 (0.7)	
Hartmann’s procedure	26 (3.9)	7 (2.3)	
No. of resected lymph nodes	30.3 ± 15.8	29.8 ± 14.9	0.636
Duration of hospital stay after surgery (day)	13.5 ± 9.4	10.3 ± 8.5	< 0.001
Postoperative morbidity	118 (17.8)	47 (15.4)	0.354
Postoperative mortality	11 (1.7)	1 (0.3)	0.082
Conversion		6 (2.0)	

Data are presented as n (%) unless otherwise indicated

**Table 3:** Pathological data

Characteristic	Open (*n* = 662)	Laparoscopy (*n* = 305)	*P*
T stage			0.009
is	8 (1.2)	8 (2.6)	
1	33 (5.0)	27 (8.9)	
2	78 (11.8)	44 (14.4)	
3	447 (67.5)	198 (64.9)	
4	96 (14.5)	28 (9.2)	
N stage			0.108
0	385 (58.2)	199 (65.2)	
1	184 (27.8)	69 (22.6)	
2	93 (14.0)	37 (12.1)	
Histology			0.886
Adenocarcinoma	618 (93.4)	287 (94.1)	
Signet ring cell	4 (0.6)	1 (0.3)	
Mucinous adenocarcinoma	36 (5.4)	16 (5.2)	
Other	4 (0.6)	1 (0.3)	
Histology grade			0.123
Well	67 (10.1)	38 (12.5)	
Moderate	530 (80.1)	248 (81.3)	
Poor	65 (9.8)	19 (6.2)	

Data are presented as n (%) unless otherwise indicated

**Fig. 1:**
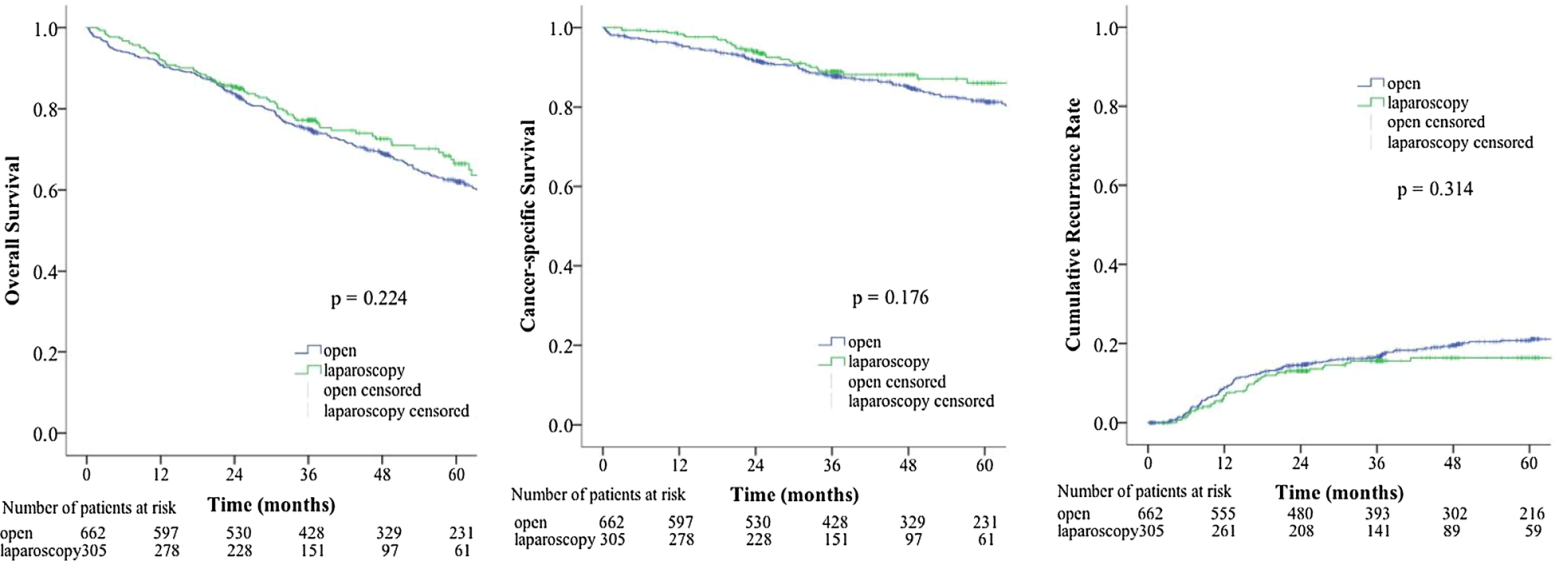
Survival rate and recurrence rates in the open and laparoscopic groups for stage 0–III colorectal cancer in elderly patients

**Fig. 2:**
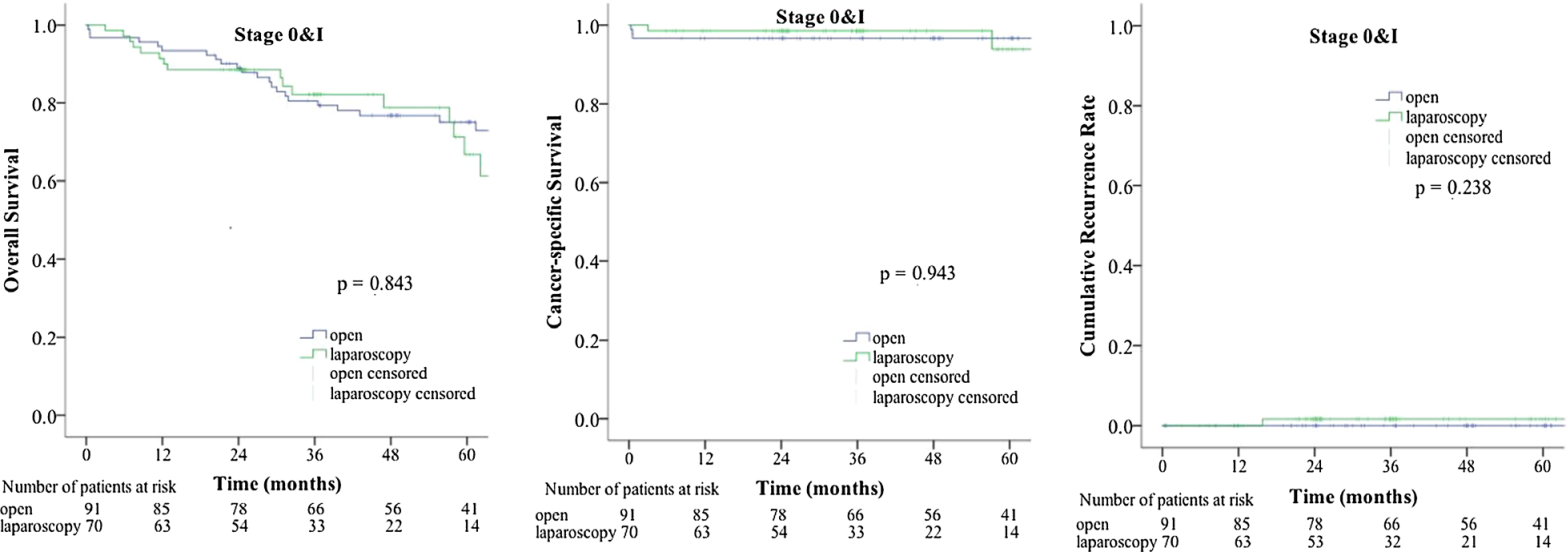
Survival rate and recurrence rates in the open and laparoscopic groups for stage 0 and I colorectal cancer in elderly patients

**Fig. 3:**
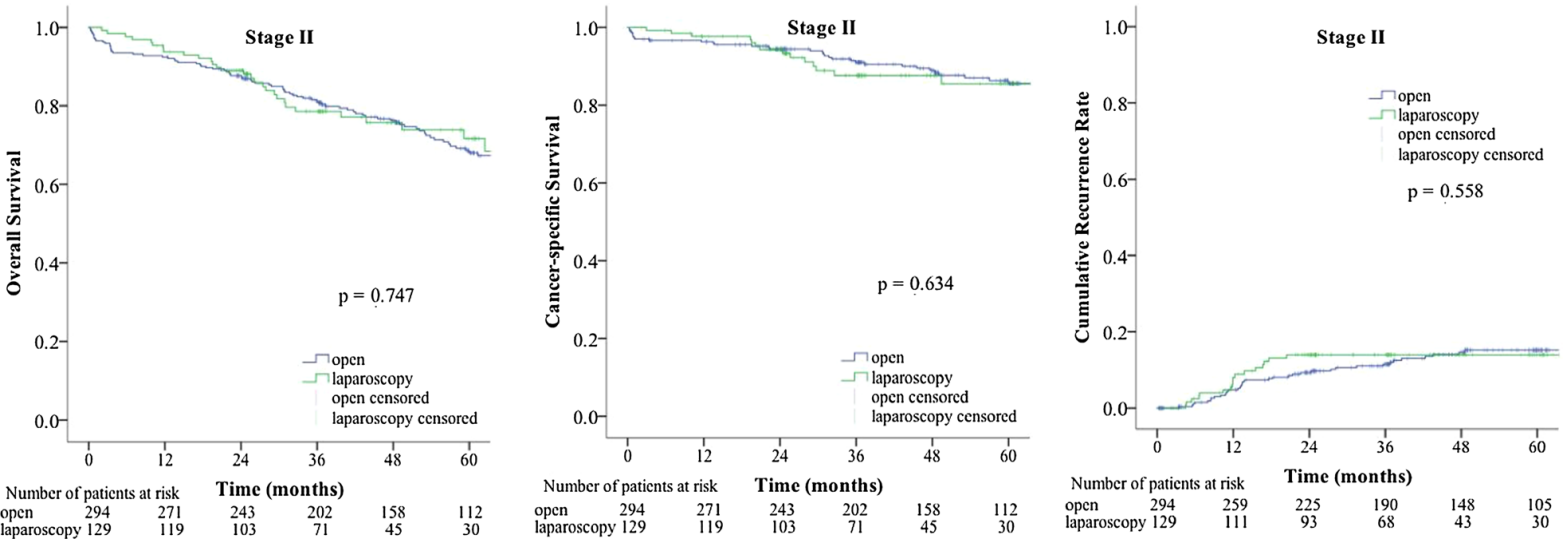
Survival rate and recurrence rates in the open and laparoscopic groups for stage II colorectal cancer in elderly patients

**Fig. 4:**
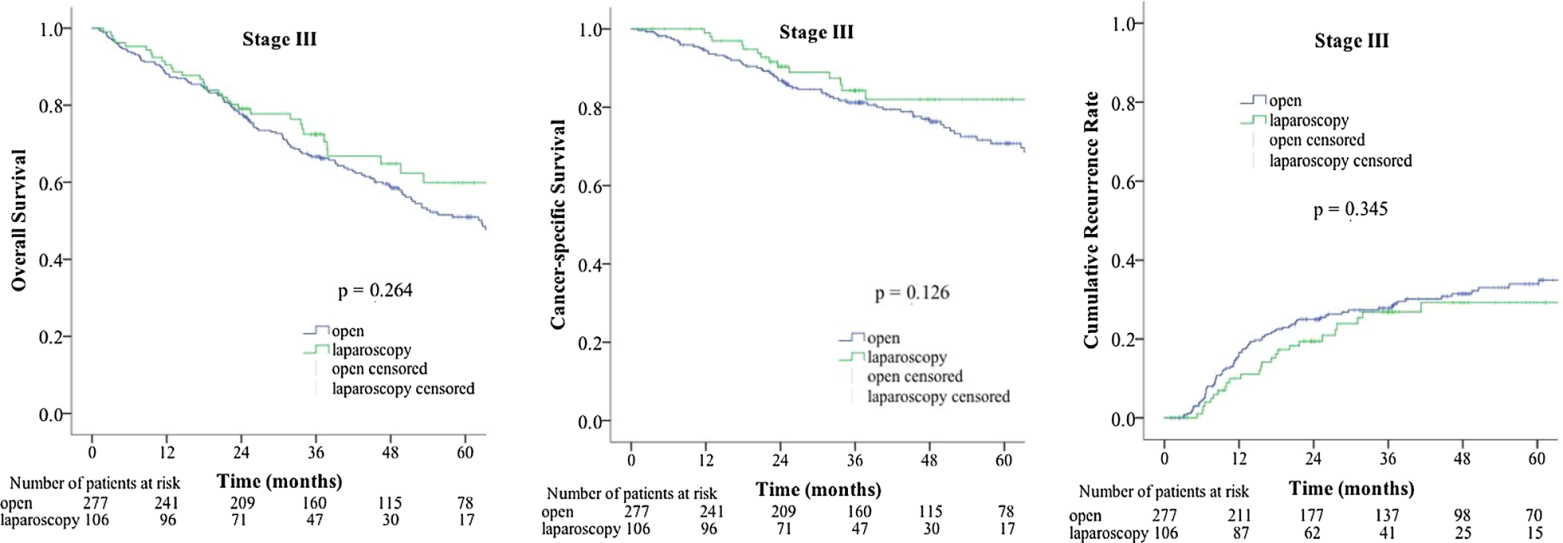
Survival rate and recurrence rates in the open and laparoscopic groups for stage III colorectal cancer in elderly patients

The median follow-up time was 47.9 months in the open group and 35.9 months in the laparoscopic group. The differences in OS, CSS, and CRR between the two groups were not statistically significant. A total of 967 patients were divided into three groups by TNM stage: the stage 0 and I group, the stage II group, and the stage III group (Fig. 1). We compared the OS, CSS, and CRR in the three groups after the open and laparoscopic surgery. In the stage 0 and I group, the OS, CSS, and CRR were similar between the open group and the laparoscopic group (Fig. 2, open vs. laparoscopic, 5-year OS rate: 75% vs. 67%, 5-year CSS rate: 97% vs. 94%, 5-year CRR: 0% vs. 2%). In the stage II group, the OS, CSS, and CRR were similar between the open group and the laparoscopic group (Fig. 3, open vs. laparoscopic, 5-year OS rate: 66% vs. 72%, 5-year CSS rate: 86% vs. 85%, 5-year CRR: 15% vs. 14%). In the stage III group, the OS, CSS, and CRR were also similar between the open group and the laparoscopic group (Fig. 4, open vs. laparoscopic, 5-year OS rate: 51% vs. 60%, 5-year CSS rate: 71% vs. 82%, 5-year-CRR: 35% vs. 29%).

## Discussion

This is a retrospective study analyzing laparoscopic radical surgery for elderly CRC patients with the largest sample size from a single academic medical center. The results presented the real-world experience in a single institute to strengthen the laparoscopic procedure for elderly patients with CRC. In our study, although elderly patients with CRC who underwent laparoscopy were prone to develop early T stage cancer, obesity, and high serum albumin level, their length of postoperative stay was significantly shorter, their postoperative morbidity and mortality were similar to that of those underwent open surgery, and the rate of conversion was acceptably low (6 out of 305 patients, and one died). In each TNM stage, the long-term outcomes including OS rate, CSS rate, and CRR between the open surgery group and the laparoscopic group were also similar.

For elderly patients with non-metastatic CRC who underwent surgery, we revealed that the overall postoperative morbidity and mortality rates were similar in both the groups. The postoperative morbidity rate was 15.4% and the mortality rate was 0.3% in the laparoscopic group. The operative mortality and morbidity of laparoscopy were similar and even lower compared to some reports [16–21], and the results represented the real-world condition about short-term outcome in Taiwan. The role of laparoscopic surgery in CRC treatment is accepted worldwide [8, 22]. However, considering the surgical risks in elderly patients (high risks of anesthesia, operative morbidity and mortality, malnutrition, immunity decline, poor performance status, and having comorbidities) [23] and long operation time, surgeons may refrain from performing laparoscopic surgery on elderly patients. Seshadri et al. reported that 62 octogenarians who received laparoscopic colorectal procedure (including benign disease) in the 1990s resulted in favorable postoperative outcome [24]. Law et al. compared the groups of open and laparoscopic colorectal resections of malignant or benign colorectal disease in Asians aged above 70 years in the early 2000, and the two groups shared similar postoperative morbidity rates [25]. Fujii et al. had reported that the postoperative complication rate in the laparoscopic group was lower than that in the open group (23% vs. 36%) for elderly patients, and the postoperative ileus rate in the laparoscopic group was significantly lower [20].

The conversion rate was 2% in our study and it was similar to or even lower than previous studies (6.1–21%) [13, 16, 26–29]. Of these converted patients, only three developed postoperative morbidity and none had tumor recurrence during follow-up. The short-term or long-term outcomes may not be influenced by the conversion if adequate surgical safety is achieved. The retrieved lymph nodes in the two groups were sufficient as suggested by the guidelines. We agreed that laparoscopic surgery could meet the similar oncological quality as open surgery for CRC treatment through this study. The length of postoperative stay was shorter in the laparoscopic group, and several studies had confirmed the result [16, 18, 25, 28, 30–32]. The most evident benefit for elderly patients to receive laparoscopy surgery is the reduced hospital stay.

To our knowledge, there were few studies focusing on the long-term oncological outcomes in elderly patients receiving open and laparoscopic surgery. This study is the largest cohort from a single medical center to analyze laparoscopic surgery for the elderly patients with CRC. The long-term oncological outcomes including OS, CSS, and CRR did not differ between the two groups. In our study, patients in the open group had much advanced TNM stage and abnormal CEA level compared to the laparoscopy group. This finding may be owing to the surgeons’ preference for patient selection. However, we divided the patients into three groups by TNM stage, and the long-term oncological outcomes showed no difference in each group. Several studies had reported that OS and disease-free survival did not differ between patients undergoing open surgery and laparoscopy [14, 31–33].

This study reports cancer-specific survival and cumulative recurrence rate, which were observed lesser in similar studies. We analyzed CSS as the elderly patients with CRC passed away not only owing to malignancy but also multiple causes related to aging, and resulting in significant reduction in OS. In this study, the CSS rate for each TNM group was about 15–30% higher than the OS rate. Hinoi and his colleague reported that the 3-year CSS rates for both colon and rectal cancer patients aged above 80 years were approximately 86.5–93.4%, similar to our result (88%) [32]. For elderly patients, once the recurrence occurred, they may not be able to tolerate recurrence treatment compared to younger patients. Cumulative recurrence was analyzed in this study for evaluating the efficacy and oncological quality of open and laparoscopic surgery. The cumulative recurrence rate was similar in each TNM stage group as mentioned in a few previous studies. According to our results, laparoscopic surgery for CRC treatment in elderly patients, could be used as a standard method for radical resection of malignancies.

The present study has some potential limitations. First, this is a retrospective study conducted at a single institute while collecting data prospectively and is subject to various biases. Second, the selection bias is an essential issue because the choice of laparoscopy or laparotomy surgery is subjective to surgeons’ preference, although the long-term outcome was compared at each stage.

## Conclusion

Laparoscopic CRC resection is suitable for elderly patients with appropriate short-term outcomes including low conversion rate and similar postoperative morbidity and mortality compared to open surgery. For long-term overall and oncological outcomes, the results of laparoscopic surgery were similar to that of open surgery in each TNM stage. Additionally, short duration of hospital stay after laparoscopic surgery is beneficial for elderly patients. Therefore, laparoscopic surgery for elderly patients with CRC can be a standard alternative for malignant treatment.

## Data Availability

The datasets used and analyzed during the current study are available from the corresponding author on reasonable request.

## Abbreviations

CRC: Colorectal cancer
BMI: Body mass index
CEA: Carcinoembryonic antigen
Hb: Hemoglobin
AST: Aspartate aminotransferase
Cr: Creatinine
OS: Overall survival
CSS: Cancer-specific survival
CRR: Cumulative recurrence rate
TNM: Tumor-node-metastasis
